# Low Seroprevalence of Bovine Brucellosis in Communal Areas of Limpopo Province, South Africa

**DOI:** 10.3390/vetsci12100942

**Published:** 2025-09-29

**Authors:** Karabelo Madiba, Nomakorinte Gcebe, Carin Boshoff, Mohamed Sirdar, Ngoako Ramaselela, Tiny Hlokwe

**Affiliations:** 1 Biotechnology Section, Diagnostic Services Program, Agricultural Research Council-Onderstepoort Veterinary Research, Onderstepoort, Pretoria 0110, South Africa; karabelo.madiba@westerncape.gov.za; 2 Bacteriology Section, Diagnostic Services Program, Agricultural Research Council-Onderstepoort Veterinary Research, Onderstepoort, Pretoria 0110, South Africa; gceben@arc.agric.za; 3 Department of Biomedical Sciences, Faculty of Science, Tshwane University of Technology, Pretoria 0083, South Africa; boshoffci@tut.ac.za; 4 Department of Production Animal Studies, University of Pretoria Faculty of Veterinary Science, Onderstepoort, Pretoria 0110, South Africa; msirdar@isid.org; 5 International Society for Infectious Diseases, Boston, MA 02116, USA; 6 Veterinary Services, Department of Agriculture, Molemole Municipality, Limpopo 0715, South Africa; ramaselelan@agric.limpopo.gov.za

**Keywords:** brucellosis, Rose Bengal Test, compliment fixation test, cattle, communal farms

## Abstract

Brucellosis is caused by Gram-negative coccobacilli of the *Brucella* genus, with cattle mainly infected with *Brucella abortus*. The disease burden is a threat to socioeconomic development (agriculture/tourism) as well as to animal health, biodiversity and to human health due to the zoonotic nature of this pathogen. In South Africa (S.A), the prevalence of the disease in cattle and livestock in general is mostly unknown in communal farms. A cross-sectional study with a multistage sampling strategy was applied in communal areas from three district municipalities, i.e., Mopani, Capricorn and Sekhukhune of Limpopo province, South Africa. Sera (*n* = 1133) were collected and screened for antibodies against the *Brucella* species using the Rose Bengal Test (RBT) and confirmation of positive reactors with a Complement Fixation Test (CFT). The brucellosis seroprevalence was found to be 0.79% (95% CI: 0.38–1.45) by a CFT. Univariate analysis indicated that only the frequency of birth was significantly associated with CFT positivity (OR = 20; 95% Cl: 1.61–247.99; *p* = 0.039). The multivariable logistic regression model revealed that the frequency of birth, age, breed, gender, municipality and district were not statistically significant predictors at 0.05 level. However, some variables like cattle aged more than five years, had higher odds of CFT positivity compared to those younger than five years (OR = 5.66; 95% CI: 0.36–87.97), although the association was not statistically significant (*p* = 0.215). All positive reactors detected originated from the Mopani district municipality. Overall, the findings reveal a much lower seroprevalence of brucellosis in the communal farms of Limpopo province than previously assumed. We are of the opinion that the low seroprevalence is attributed to effective control strategies implemented by the Limpopo provincial veterinary services and hence provide important information to assist the regulatory bodies in the control and eradication of the disease.

## Simple Summary

Brucellosis is a disease that affects both animals and people, mainly spread through infected cattle, and it can harm farming and public health. In South Africa, little is known about the occurrence of this disease in cattle kept on communal farms. This study looked at cattle from three districts in Limpopo province to find out how many cattle had been exposed to the brucella species. Blood samples from over one thousand cattle were tested, and the results showed that less than 1% of the animals carried signs of the disease. Most positive reactors came from one district only. The findings suggest that brucellosis is much less common in Limpopo’s communal cattle than previously thought. Knowing that the level of infection is low is important for farmers, veterinarians and policymakers, as it means current strategies are working, but continued monitoring is needed. These results help protect both animal and human health, support safe food production and contribute to the wellbeing of rural communities in South Africa.

## 1. Introduction

Brucellosis caused by Brucella bacteria is a highly infectious and contagious zoonotic disease that dates back at least to 750 BC [1]. The six classical species of the Brucella genus include *Brucella melitensis*, *Brucella abortus*, *Brucella canis*, *Brucella ovis*, *Brucella suis* and *Brucella neotomae*. The six non-classical species are *Brucella ceti*, *Brucella microti*, *Brucella inopinata*, *Brucella vulpis*, *Brucella papionis* and *Brucella pinnipedialis* [2]. The most common symptom of brucellosis in animals is normally abortion in first pregnancy after infection; however, the animal becomes less likely to abort thereafter [3]. Brucellosis has long been reported in South Africa, and the first documented cases were of *B. melitensis* in sheep in the former Transvaal Province in 1965 (now known as Gauteng, Limpopo, Mpumalanga and the eastern parts of Northwest Province) [4]. Brucellosis is a notifiable disease in South Africa, and all suspected and confirmed cases of abortions must be reported to the nearest State Veterinary office for zoo-sanitary actions as prescribed in the National Brucellosis Control Scheme [5]. Worldwide, there are three known vaccines, namely the strains *Brucella abortus* S19, *Brucella abortus* RB51 for cattle and *Brucella melitensis* Rev1 for sheep and goats, which are used to control the disease [6]. In South Africa, *Brucella abortus* S19 is used in cattle calves and *Brucella abortus* RB51 for adult cattle [7]. The disease burden is a threat to socioeconomic development (agriculture/tourism) as well as to animal health, biodiversity and human health, due to the zoonotic nature of this pathogen. Moreover, rural communities are economically and culturally dependent on livestock and there is always unavoidable close and frequent contact between human and animals [8,9]. It is also a concern that through consumption of contaminated milk and meat, both livestock owners and the wider community might be at a high risk of acquiring the disease. It is thus critical to determine the prevalence and associated risk factors to understand the degree and severity of the problem to be able to suggest possible intervention strategies [9].

Limited information is available on the prevalence of brucellosis in rural communities practicing communal livestock management systems in South Africa [10]. An adequate record on the status of brucellosis in livestock in the communal and emerging production systems in Limpopo Province, South Africa, is lacking. Apart from a study conducted in Mnisi, Limpopo Province (South Africa) by Simpson and co-authors [7], there are no reports on the seroprevalence of brucellosis in communal cattle of Limpopo Province; hence, the ultimate goal of this study was to produce comprehensive data on the seroprevalence of bovine brucellosis in communal farms of Limpopo Province of South Africa.

## 2. Materials and Methods

### 2.1. Study Design and Sampling Sites

**Figure 1:**
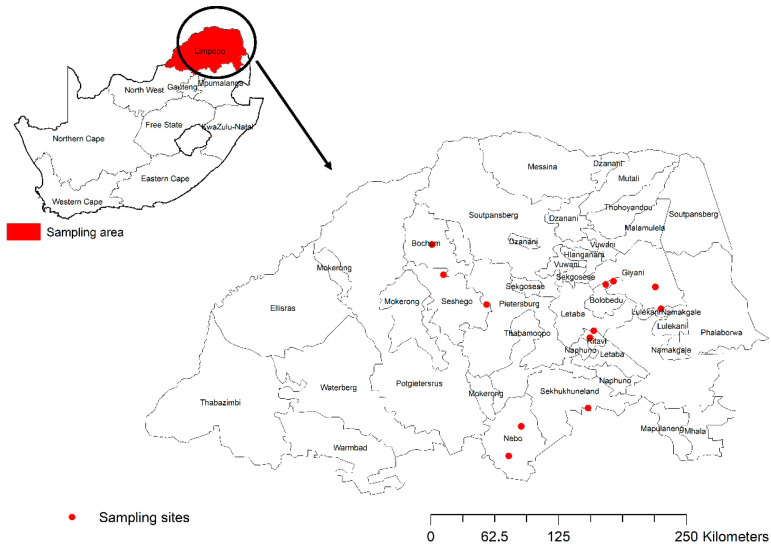
Map of South Africa indicating Limpopo province and sampling area in red dots.

This was a cross-sectional study in which stratified random sampling was used for sampling in the communal areas of Limpopo Province, South Africa (Figure 1). Sampling was performed between May and November 2018 and included three of the five districts in the province. Cattle above the age of one year were randomly sampled at dip tanks (animal assembly points) during each sampling visit. Areas with significant mining activities, commercial livestock production systems and farms bordering the foot-and-mouth disease (FMD) protection areas were excluded from the study. Animal demographic data such as age, gender, breed, birth history, district and local municipality were obtained from the animal owners.

### 2.2. Sample Size Determination and Sampling

A formula that estimates sample size for determining population proportion was used.

$$n=\frac{1.96^{2\;}p(1-p)}{d^{2}}$$

*p* = estimated prevalence, *d* = desired absolute precision at a type 1 error of 0.045, *n* = estimated sample size.

A 50% prevalence is often used when there is no background data since it provides the highest sample size. Considering that there is no current data on the prevalence of brucellosis in livestock in Limpopo Province of South Africa, an estimated prevalence of 50% and a precision of 4.5%, i.e., 0.045 was used, giving a required minimum sample size of 475. Cattle population from different local municipalities under three districts were sampled at the time of the site visits at the village dip tanks (animal assembly points). During routine dipping, animals were restrained using crush pens for a short period to allow for sample collection in the respective areas.

### 2.3. Blood Sample Collection

Venous blood was drawn from the coccygeal vein. A total of 5–7 mL of blood was collected in vacutainer blood tubes (ThermoFisher Scientific, Pretoria, South Africa) and placed in an upright position to allow for clotting to occur. The samples were kept in a fridge at 4 °C before transportation to the laboratory at the Agricultural Research Council-Onderstepoort Veterinary Research (ARC-OVR). Once in the laboratory, samples were centrifuged at 1500× *g* for 15 min and serum was harvested and stored at 4 °C (short-term storage for a week) or −20 °C (long-term storage for more than 1 week to several months) until analysis [11]. The serum samples were first screened for the presence of *Brucella* antibodies using the Rose Bengal blood test (RBT). A compliment fixation test was used as a confirmatory test [11].

### 2.4. Rose Bengal Blood Test (RBT)

The RBT is considered to be a suitable screening test for brucellosis [11]. Serum was screened for antibodies against *Brucella abortus* using the RBT, following a standardised procedure based on the World Organisation for Animal Health (WOAH) Terrestrial Manual [11]. The antigen and positive *B. abortus* control sera were obtained from Onderstepoort Biological Products, Pretoria, South Africa and an in-house negative control serum was used. The presence of *Brucella* antibodies is indicated by the presence of agglutination with a definite rim and some clearing, whereas the absence of *Brucella* antibodies was indicated by the absence of agglutination.

### 2.5. Compliment Fixation Test (CFT)

The complement fixation assay is a widely used confirmatory test for brucellosis and has been standardised [12]. The compliment fixation test was performed on samples with RBT positive results as previously described by [12,13]. The test was performed in two stages. The first stage involved an antigen mixed with the complement. In the second stage, sheep red blood cells mixed with anti-sheep antibodies were added. If the complement had been fixed in the first stage, no haemolysis took place. The results were read using a magnifying mirror. If heamolysis took place, the result was recorded as negative. If there was no haemolysis, the result was recorded as positive.

### 2.6. Statistical Analysis

Data obtained from the sample collection and serological testing was recorded in a Microsoft Excel ^®^spreadsheet. Descriptive analysis for seroprevalence was expressed in percentages (%) with a 95% confidence interval (CI) and was performed using open-source epidemiologic statistics for public health (OpenEpi) [14].

The univariate association of animal age, breed, birth history and district municipality with brucellosis positivity was determined using results obtained from a positive RBT. The odds ratio was reported as the measure of association between prevalence and possible risk factors. A 95% confidence interval and a *p* value < 0.05 were considered significant. All potential risk factors were evaluated using multivariable binary logistic regression, using results obtained from a positive CFT as a dependent variable. In the multivariable logistic regression analysis, the full model was first fitted with the variables that were significant at univariable analysis. Variables were then removed, one at a time, and their significance was assessed using the Wald statistic. Statistical modelling was performed using commercial software (IBM SPSS Statistics Version 29, International Business Machines Corp., Armonk, NY, USA).

### 2.7. Ethical Statement

Two Animal Ethics Committees approved this study: the Agricultural Research Council-Onderstepoort Veterinary Research (AEC 18/17) and Tshwane University of Technology (AREC2019/03/008) committees. Permission to undertake the study was also granted by the Department of Agriculture, Land Reforms and Rural Development (DALRRD) through Section 20 approval, in terms of the Animal Diseases Act No 35 of 1984 (12/11/1/1(729).

## 3. Results

A total of 1133 samples were collected from cattle in Capricorn, Mopani and Sekhukhune district municipalities (Figure 1). The majority of sampled animals were females (*n* = 1103; 97.35%) and the minority were bulls (*n* = 30; 2.65%). The most sampled breed was Nguni (*n* = 581; 51.42%) followed by unidentified breed (*n* = 380; 33.53%), then mixed breed (*n* = 126; 11.12%) and the least sampled breed was Brahman (*n* = 46; 4.06%).

### 3.1. Seroprevalence by RBT

Antibodies against *Brucella* spp. were detected in 48 (4.24%; CI: 95%: 3.18–5.23) cattle originating from Letaba (*n* = 21; 9.38%; CI: 95%: 6.05–13.74) and Tzaneen (*n* = 27; 4.51%; CI: 95%: 3.05–6.40) local municipalities in the Mopani district municipality. Only positive reactor variant groups were further analysed. Overall, only cows reacted positively to antibodies against *Brucella* spp. (4.35%; 95% CI: 3.26–5.68). The positive reactor breeds were from the Nguni breed (4.65% 95% CI: 3.21–6.72), mixed breed (8.73%; 95% CI: 4.87–15.25), Brahman (13.04%; 95% CI: 5.59–25.68) and unidentified (1.05%; 95% CI: 0.35–2.61). The positive reactor age groups included cows which were more than five years old (5.22%; 95% CI: 3.48–7.49), those that were less than five years old, (3.52%; 95% CI: 2.29–5.15) and heifers (8.63%; 95% CI: 4.76–14.21). Additionally, cows that had given birth less than twice (3.85%; 95% CI: 2.155–6.326), those that had given birth more than twice (7.34%; 95% CI: 4.41–11.14) and those with undefined birth status (1.74% 95% 95% CI: 0.76–3.41) also reacted positively. Cows that were expecting (*n* = 2) and those that had a history of abortion (*n* = 4) were negative (Table 1).

### 3.2. Seroprevalence by CFT

Only nine samples reacted positively to a CFT, resulting in an overall seroprevalence of 0.79% (95% CI: 0.38–1.45). All brucellosis confirmed to be seropositive were cows from the Mopani district (Tzaneen, *n* = 5 and Letaba, *n* = 4), including Nguni (*n* = 5), mixed breed (*n* = 2) and unidentified breed (*n* = 2) with no history of abortion (Table 2).

**Univariate and multivariable logistic regression analysis**

The association between CFT seropositivity and potential risk factors was analysed using univariate and multivariable logistic regression. Univariate analysis indicated that only “Frequency of birth” was significantly associated with CFT positivity (OR = 20; 95% Cl: 1.61–247.99; *p* = 0.039), whereas age, breed, gender, municipality and district were not statistically significant predictors at 0.05 level. The multivariable logistic regression revealed that none of the predictors showed any statistical significance at 0.05 level; however, some variables like cattle aged more than five years had higher odds of CFT positivity compared to those younger than five years (OR = 5.66; 95% CI: 0.36–87.97), although the association was not statistically significant (*p* = 0.215). The main limitation of this analysis is the small sample size which may have affected statistical power.

## 4. Discussion and Conclusions

Communal livestock farming is one of the world’s oldest farming systems and is typically practised by communal households in developing countries, especially in Africa [15]. The objective of this study was to determine the seroprevalence of brucellosis in cattle from communal farms in Limpopo province, South Africa. The seroprevalence of the disease was found to be very low, at 0.79% (95% CI: 0.38–1.45) using a CFT. Our results aligned with a study conducted in 2017, in the Mnisi community (Limpopo province), an area bordering wildlife reserves with multiple wildlife species infected with brucellosis, which also reported a low seroprevalence of 1.4% in cattle [7]. Contrary, a previous retrospective study by [1], between the years 2007 and 2015, reported a higher seroprevalence of 19.67% in cattle originating from both commercial and communal farms in Limpopo province, using a CFT. The study used a reference of nine years, whereas in our study, we analysed samples collected in one year, from cattle only in dip tank sites (communal). The difference in study periods, as well as the different farming systems, i.e., commercial and communal [1] versus only communal from the current study, as well as the study period, may have contributed to the differences in the seroprevalence of the disease. Improved control strategies and bio-security measures may also have been affected over time in communal farms of Limpopo province, resulting in reduced spread of the disease [16]. In comparison to other provinces of South Africa, the brucellosis seroprevalence in Limpopo province’s communal cattle was lower than that found in KwaZulu-Natal (KZN) province (1.45%) between the years 2001 and 2003 [10] as well as in a recent study by Marumo and co-workers [17], which reports a seroprevalence of 1.95% in communal and smallholder, as well as slaughter cattle, using a CFT. Contrary, in Northwest province, a relatively higher seroprevalence of 5.19% was reported in communal cattle between the years 2009 and 2013 [18].

Although our study was conducted in only one region of the country, i.e., Limpopo province, the recorded seroprevalence was lower than that observed in neighbouring countries such as Zimbabwe, where a seroprevalence of 16.7% was recorded in communal cattle using the RBT and confirmed by a CFT [19]. Similarly, a higher seroprevalence of 7.53% was recorded in cattle from Zambia using the RBT as a screening test and Competitive ELISA as a confirmatory test [20]. In Kenya, the seroprevalence was recorded at 12.44%, using an indirect enzyme linked immunosorbent assay (i-ELISA). The study was conducted in an area closer to the wildlife reserve and no suitable control measures were implemented at the time of the study, which could have contributed to the high seroprevalence [21]. As compared to other African countries, control strategies for bovine brucellosis in South Africa, which include vaccination of calves as well as voluntary testing of cattle, seem to have had a positive outcome by lowering the prevalence of disease in communal settings in Limpopo, Northwest and KZN provinces [7,17,18]. However, in this study, there is no evidence of vaccination of cattle within the study population. This statement is supported by the results of a questionnaire administered to the owners of cattle in the same study area, where only 12% (4 out of 79) reported awareness of the existence of a *Brucella* vaccine. Furthermore, an official report by the Department of Agriculture indicated that in general, there is poor compliance with brucellosis vaccination and testing [5].

In the current study, all 4.24% (95% CI: 3.18–5.23) of cattle with antibodies against *Brucella* spp. detected using the RBT, and 0.79% (95% CI: 0.39–1.45) CFT positive reactors were cows. This could have been attributed to the fact that more cows (97.35%, 95% CI: 96.29–98.17) than bulls (2.65%, 95% CI: 1.83–3.11) were sampled.

The two districts with histories of cattle abortions (0.34%, 95% CI: 0.1–0.83) did not have positive reactors. Our findings are in contrast to a study that showed abortions to be a risk factor for brucellosis infection [22]. It should, however, be noted that abortions in cattle could be due to other reproductive diseases such as Q-fever [23]. Univariate analysis indicated that only “Frequency of birth” was significantly associated with CFT positivity (OR = 20; 95% Cl: 1.61–247.99; *p* = 0.039), whereas age, breed, gender, municipality and district were not statistically significant predictors at 0.05 level. Although the multivariable logistic regression analysis revealed that none of the predictors showed any statistical significance at 0.05 level, variables such as cattle aged over five years showed high odds ratios, suggesting a potential association with CFT positivity (OR = 5.66; 95% CI: 0.36–87.97); the association was not statistically significant (*p* = 0.215). Almost like the outcome of this study, research conducted in Western Ethiopia found that the majority of the older cattle group tested positive for brucellosis [24]. Our findings are contrary to the outcome of the previous study conducted in this province, where no association between gender and seropositivity for brucellosis was found [7]. Similarly, a study conducted in Cameroon also showed no association between sex in relation to Brucella infections [25]. Likewise, another study conducted in Niger showed no association between sex and seropositivity [26].

In conclusion, we report a very low seroprevalence of brucellosis in communal cattle from Limpopo province of South Africa. It should be noted that both RBTs and CFTs cannot differentiate amongst *Brucella* species; therefore, the detected antibodies indicate exposure to *Brucella* species but not necessarily to *Brucella abortus* specifically. We are of the opinion that the low seroprevalence is attributed to effective control strategies implemented by the Limpopo provincial veterinary services.

## 5. Limitations to the Study

Samples were collected at dip-tank level, and it was not possible to trace an individual animal to a specific cattle herd. Individuals could not be grouped by farm of origin or herd. Clustering in sample size calculation and subsequent analysis could not be accounted for. The other option could have been to account for clustering at the municipality level; however, the outcome could have been too broad to generalise at this level. Additionally, animals classified as an ‘unidentified breed’ were unfortunately not included in the analysis. These were those animals whose information regarding their breed was not provided at the dip tanks, and there were no records of the breeds. Furthermore, the age categorization of the animals sampled was too broad. Unfortunately, it was not possible to obtain more accurate age responses from farmers, since these communal farmers do not keep animal records.

Although the vaccination history was not provided, we acknowledge that if animals were vaccinated, it might have impacted the outcome of the analysis. The small sample size may have affected the statistical power of the univariate and multivariable logistic regression analysis.

## Figures and Tables

**Table 1:** Seroprevalence by RBT of bovine brucellosis in Limpopo province, South Africa during May–November 2018.

Variable	Samples Collected and Tested for RBT (Positive Reactor Group)	RBT Positive (No.)	Sero Prevalence % 95% CI
**District municipality**	Mopani (*n* = 823)	48	5.83 (4.38–7.59)
	Capricorn (*n* = 167)	0	0.00 (0.00–0.26)
	Sekhukhune (*n* = 143)	0	0.00 (0.00–0.26)
**Local municipality**	Tzaneen (*n* = 599)	27	4.51 (3.05.6.40)
	Letaba (*n* = 224)	21	9.38 (6.05–13.74)
	Blouberg (*n* = 100)	0	0.00 (0.00–2.95)
	Ephraim Mogale (*n* = 43)	0	0.00 (0.00–6.73)
	Makhuduthamaga (*n* = 100)	0	0.00 (0.00–2.95)
	Polokwane (*n* = 67)	0	0.00 (0.00–4.37)
**Sex**	Female (*n* = 1103)	48	04.35 (3.26–5.68)
	Male (*n* = 30)	0	0.00 (0.00–0.26)
**Age**	>5 years (*n* = 479)	25	5.22 (3.48–7.49)
	<5 years (*n* = 654)	23	3.52 (2.29–5.15)
**Breed**	Nguni (*n* = 581)	27	4.65 (3.21–6.72)
	Mixed (*n* = 126)	11	8.73 (4.87–15.25)
	Unidentified (*n* = 380)	4	1.05 (0.35–2.61)
	Brahman (*n* = 46)	6	13.04 (5.59–25.68)
**Birth history**	Heifer (*n* = 139)	12	8.63 (4.76–14.21)
	<2 times (*n* = 338)	13	3.85 (2.155–6.326)
	>2 times (*n* = 218)	16	7.34 (4.41–11.14)
	Unidentified (*n* = 402)	7	1.74 (0.76–3.41)
	Abortion (*n* = 4)	0	0.00 (0.10–0.83)
	Expecting (*n* = 2)	0	0.00 (0.10–0.83)
	Male (*n* = 30)	0	0.00 (0.10–0.83)

**Table 2:** The distribution/demographic and herd-related characteristics for Brucellosis-positive cattle using the Compliment Fixation Test (CFT) (*n* = 9).

Variable	Category	No. of Positive Animals	(%)
**Frequency of birth**	Not indicated Heifer Less than 2 More than 2	4 2 2 1	44.40 22.20 22.20 11.10
**Age**	More than 5 Less than 5	6 3	66.70 33.30
**Breed**	Nguni Mix Not indicated Brahman	5 2 20	55.60 22.20 22.20 00.00
**Gender**	Male Female	09	0.00 100
**Municipality**	Tzaneen Letaba	5 4	55.60 44.40
**District**	Mopani	9	100

## Data Availability

The datasets generated and analysed during the current study are not publicly available due to the privacy of research participants but are available from the corresponding author on reasonable request.

## References

[1] Kolo F.B., Adesiyun A.A., Fasina F.O., Katsande C.T., Dogonyaro B.B., Potts A., Matle I., Gelaw A.K., Van Heerden H. (2019). Seroprevalence and characterization of Brucella species in cattle slaughtered at Gauteng abattoirs, South Africa. Vet. Med. Sci..

[2] Matle I., Ledwaba B., Madiba K., Makhado L., Jambwa K., Ntushelo N. (2021). Characterisation of Brucella species and biovars in South Africa between 2008 and 2018 using laboratory diagnostic data. Vet. Med. Sci..

[3] Ducrotoy M., Bertu W.J., Matope G., Cadmus S., Conde-Álvarez R., Gusi A.M., Welburn S., Ocholi R., Blasco J.M., Moriyón I. (2017). Brucellosis in Sub-Saharan Africa: Current challenges for management, diagnosis and control. Acta Trop..

[4] Van Drimmelen G.C. (1965). The presence of *Brucella melitensis* infection in sheep in the Transvaal. Bull. I’Office Int. Ds Epizoot..

[5] Department of Agriculture Forestry & Fisheries (DAFF) (2017). Discussion Paper on the Review of Bovine Brucellosis Control in South Africa.

[6] Barbosa A.A., Figueiredo A.C.S., Palhao M.P., Viana J.H.M., Fernandes C.A.C. (2017). Safety of vaccination against brucellosis with the rough strain in pregnant cattle. Trop. Anim. Health Prod..

[7] Simpson G., Marcotty T., Rouille E., Matekwe N., Letesson J.J., Godfroid J. (2018). Documenting the absence of brucellosis in cattle, goats and dogs in a “One Health” interface in the Mnisi community, Limpopo, South Africa. Trop. Anim. Health Prod..

[8] Godfroid J. (2002). Brucellosis in wildlife. OIE Rev. Sci. Tech.-Off. Int. Epizoot..

[9] Muendo E.N., Mbatha P.M., Macharia J., Abdoel T.H., Janszen P.V., Pastoor R., Smits H.L. (2012). Infection of cattle in Kenya with Brucella abortus biovar 3 and Brucella melitensis biovar 1 genotypes. Trop. Anim. Health Prod..

[10] Hesterberg U.W., Bagnall R., Perrett K., Bosch B., Horner R., Gummow B. (2008). A serological prevalence survey of Brucella abortus in cattle of rural communities in the province of KwaZulu-Natal, South Africa. J. S. Afr. Vet. Assoc..

[11] WOAH (2018). Chapter 3.1.4 Brucellosis (Brucella abortus, B. melitensis and B. suis (Infection with B. abortus, B. melitensis and B suis). Terrestrial Animal Health Code: Recommendations Applicable to OIE Listed Diseases.

[12] Hill W. (1963). Standardization of the complement fixation test for brucellosis. Bull. OIE.

[13] Herr S., Huchzermeyer H.F., Te Brugge L.A., Williamson C.C., Roos J.A., Schiele G.J. (1985). The use of a single complement fixation test technique in bovine brucellosis, Johne’s disease, dourine, equine piroplasmosis and Q fever serology. Onderstepoort J. Vet. Res..

[14] https://www.openepi.com/Proportion/Proportion.htm.

[15] Mmbengwa V., Nyhodo B., Myeki L., Ngethu X., van Schalkwyk H. (2015). Communal livestock farming in South Africa: Does this farming system create jobs for poverty stricken rural areas. Sylwan.

[16] Government Gazette Republic of South Africa. 2002. Animal Health Act No. 23675, Volume 445, Cape Town. 30 July 2002. https://www.gov.za/sites/default/files/gcis_document/201409/a7-02.pdf.

[17] Marumo B., Hlokwe T.M., Kayoka-Kabongo P.N. (2023). Seroprevalence of brucellosis in communal and smallholder cattle farming in Northwest Province, South Africa. Onderstepoort J. Vet. Res..

[18] Harris B., Manoto S.N., McCrindle C.M. (2020). Sero-prevalence of bovine brucellosis in the Bojanala Region, Northwest Province, South Africa 2009–2013. J. S. Afr. Vet. Assoc..

[19] Ndengu M., Matope G., de Garine-Wichatitsky M., Tivapasi M., Scacchia M., Bonfini B., Pfukenyi D.M. (2017). Seroprevalence of brucellosis in cattle and selected wildlife species at selected livestock/wildlife interface areas of the Gonarezhou National Park, Zimbabwe. Prev. Vet. Med..

[20] Mfune R.L., Mubanga M., Silwamba I., Sagamiko F., Mudenda S., Daka V., Godfroid J., Hangombe B.M., Muma J.B. (2021). Seroprevalence of bovine brucellosis in selected districts of Zambia. Int. J. Environ. Res. Public Health.

[21] Enström S., Nthiwa D., Bett B., Karlsson A., Alonso S., Lindahl J.F. (2017). Brucella seroprevalence in cattle near a wildlife reserve in Kenya. BMC Res. Notes.

[22] Deresa B., Tulu D., Deressa F.B. (2020). Epidemiological investigation of cattle abortion and its association with Brucellosis in Jimma Zone. Vet. Med. Res. Rep..

[23] Mangena M., Gcebe N., Pierneef R., Thompson P.N., Adesiyun A.A. (2021). Q fever: Seroprevalence, risk factors in slaughter livestock and genotypes of *Coxiella burnetii* in South Africa. Pathogens.

[24] Tolosa T., Regassa F., Belihu K. (2008). Seroprevalence study of bovine brucellosis in extensive management system in selected sites of Jimma Zone, Western Ethiopia. Bull. Anim. Health Prod. Afr..

[25] Bayemi P.H., Webb E.C., Nsongka M.V., Unger H., Njakoi H. (2009). Prevalence of *Brucella abortus* antibodies in serum of Holstein cattle in Cameroon. Trop. Anim. Health Prod..

[26] Boukary A.R., Saegerman C., Abatih E., Fretin D., Bada R.A., De Deken R., Harouna H.A., Yenikoye A., Thys E. (2013). Seroprevalence and potential risk factors for *Brucella* spp. infection in traditional cattle, sheep and goats reared in urban, periurban and rural areas of Niger. PLoS ONE.

